# Ex vivo porcine model study on the treatment outcomes of scissor-type knife versus needle-type knife in endoscopic submucosal dissection performed by trainees

**DOI:** 10.1186/s12893-020-00955-w

**Published:** 2020-11-19

**Authors:** Ryoji Ichijima, Mitsuru Esaki, Shun Yamakawa, Yosuke Minoda, Sho Suzuki, Chika Kusano, Hisatomo Ikehara, Takuji Gotoda

**Affiliations:** 1 Division of Gastroenterology and Hepatology, Department of Medicine, Nihon University School of Medicine, 1-6 Kanda-Surugadai, Chiyoda-ku, Tokyo, 101-0062 Japan; 2 Department of Medicine and Bioregulatory Science, Graduate School of Medicine Sciences, Kyushu University, Fukuoka, Japan

**Keywords:** Endoscopic submucosal dissection, Endo-knife, Scissor-type, Porcine model, Ex vivo

## Abstract

**Background:**

Endoscopic submucosal dissection (ESD) for gastrointestinal neoplasms can be technically difficult for trainee endoscopists. Presently, there is no consensus for trainees to select the endo-knife type in ESD. Therefore, we conducted a comparison study of treatment outcomes between scissors-type and needle-type knives in ESD performed by trainees in an ex vivo porcine model.

**Methods:**

This study was conducted on trainee endoscopists who participated in ESD hands-on seminars held in August 2018 and September 2019. A total of 22 trainees from 13 institutions were divided into two groups according to their endoscopic experience. Under expert supervision, each trainee performed two ESDs in porcine models, namely, scissor-type knife (ESD-S) and needle-type knife (ESD-N). The efficacy and safety, including the procedure time and rates of self-completion, en bloc resection, and complications, were compared between ESD-S and ESD-N. In subgroup analysis, we also investigated the predictors associated with the difficulty of ESD for trainees using multivariate logistic regression analysis.

**Results:**

Eight trainees had an experience of over 1000 endoscopies (senior trainee: S-Trainee), whereas the others had an experience of less than 1000 endoscopies (junior trainee: J-Trainee). Among the S-Trainees, no significant differences were observed in any treatment outcome between ESD-S and ESD-N. Among the J-Trainees, the total procedure and mucosal incision times were significantly shorter in ESD-S than in ESD-N [total procedure time: 16.5 min (range 10.0–31.0) vs. 22.3 min (range 10.0–38.0), P = 0.018; circumferential incision time: 10.0 min (range 6–16) vs. 17.0 min (range 5.0–31.5); P = 0.019]. Regarding complications, muscular injury occurred in two patients during ESD-N performed by J-Trainees; however, no muscular injury occurred during ESD-S. In subgroup analysis, ESD-N was an independent predictive factor of difficult ESD (odds ratio 5.28, 95% confidence interval 1.25–22.30; *P* = 0.024).

**Conclusions:**

This study revealed that trainees, particularly those who have experienced less than 1000 endoscopies, should opt for the scissor-type knife to perform ESD.

## Background

Endoscopic submucosal dissection (ESD) is widely accepted as a less invasive treatment for early gastrointestinal neoplasms [1, 2]. Developed in Japan during the 1990s, ESD has since undergone various advancements [3, 4] that have decreased the complication rates and increased en bloc resection rates [5–7]. However, it remains a difficult technique for trainees to perform independently. The self-completion rate of ESD for early gastric neoplasms, when performed by trainees and supervised by expert endoscopists, is only 60% [8].

A variety of endo-knives have been developed and used for ESD [9–11]. Generally, the type of endo-knife used differs with each institution. Therefore, only a few reports are available that compare the treatment outcomes of ESD according to the endo-knives used in the procedure [11–13]. There is no consensus on which endo-knife is the most suitable for ESD because favorable treatment outcomes have been reported for each endo-knife. However, the treatment outcomes of ESD performed by trainees are unsatisfactory; we speculate that the type of endo-knife used may affect treatment outcomes because the techniques of the trainees are generally immature.

Hands-on training for ESD under expert supervision in a porcine model is recommended for trainees before performing ESD on patients [12–16]. There has been no study comparing different endo-knives in the treatment outcomes of ESD performed by trainees in a porcine model. Therefore, in this study, using a porcine model, we compared the treatment outcomes of ESD between the scissor-type and needle-type endo-knives in terms of their efficacy and safety.

## Methods

### Study design

**Fig. 1:**
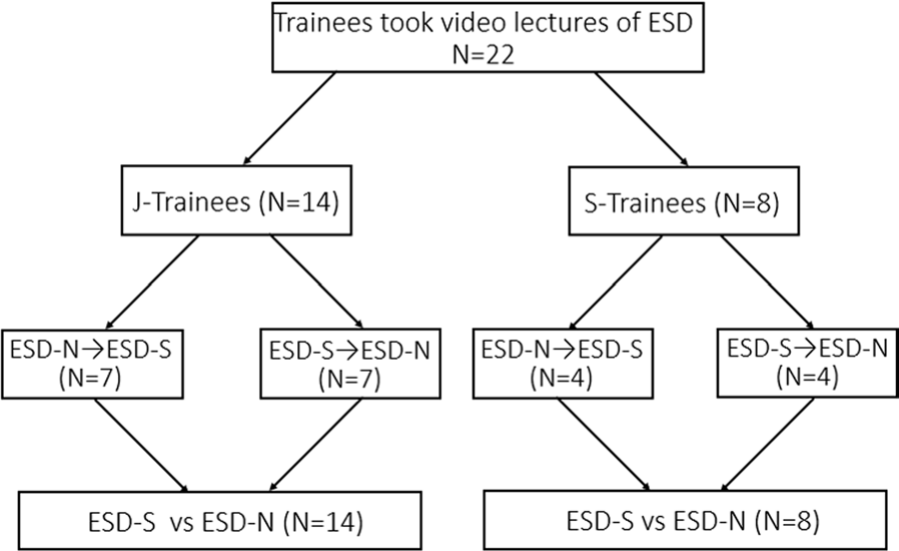
Flow chart demonstrating the categorization of the included trainees. *J-Trainee* junior-trainee with experience less than 1000 endoscopies, *S-Trainee* senior-trainee with an experience of more than 1000 endoscopies, *ESD-N* endoscopic submucosal dissection with a needle-type knife, *ESD-S* endoscopic submucosal dissection with a scissor-type knife

This study was conducted among trainee endoscopists who participated in ESD hands-on seminars held in August 2018 and September 2019 at the Kitakyushu Municipal Medical Center. A total of 22 trainees from 13 institutions performed ESD on porcine models; 20 trainees from 11 institutions in Japan and 2 trainees from 2 institutions in China. Before the ESD training, all trainees reviewed video lectures on ESD procedures with scissor-type and needle-type endo-knives. Thereafter, each trainee performed two ESDs under expert supervision, one using the scissor-type knife (ESD-S) and the other using the needle-type knife (ESD-N). Owing to differences in experience among the trainees, they were classified into two groups according to their experience in upper or lower gastrointestinal (GI) endoscopy. One group included 14 trainees who had experienced less than 1000 upper or lower GI endoscopies (junior-trainee: J-Trainee), while the other group included eight trainees who had experienced more than 1000 endoscopies (senior trainee: S-Trainee). Within each group, the trainees were further randomly assigned to two additional groups: ESD-S and ESD-N; after performing ESD with one knife, they were reassigned to the other group in which they performed with the other knife (Fig. 1). All ESDs were performed under expert assistance. One expert and one trainee were assigned to each endoscopy room, where the experts supervised and advised the trainees during the ESD procedure. After the completion of all procedures, the outcomes were recorded in a case report form.

The study protocol was approved by the Institutional Review Board of the Kitakyushu Municipal Medical Center (No. 201901076). The requirement for written informed consent was waived because this was an ex vivo porcine model study; all trainees were orally informed about the study and consented to participate. This study was registered as a retrospective study with the University Hospital Medical Information Network (UMIN) Clinical Trials Registry (www.umin.ac.jp/ctr/) (UMIN00041827). Although this was an ex vivo animal model study, it was conducted in accordance with the guidelines of the Animal Research Reporting In Vivo Experiments (ARRIVE) as much as possible.

### Trainees and experts

Trainees who had performed more than 100 upper gastrointestinal (GI) endoscopies but no or less than five ESDs (performed independently without self-completion), were included in this study. All experts were endoscopy specialists certified by the Japan Gastroenterological Endoscopy Society and had performed more than 100 ESDs. Experts familiar with the scissor-type knife supervised the ESD-S procedure, while those familiar with needle-type knife supervised the ESD-N procedure.

### Devices

**Fig. 2:**
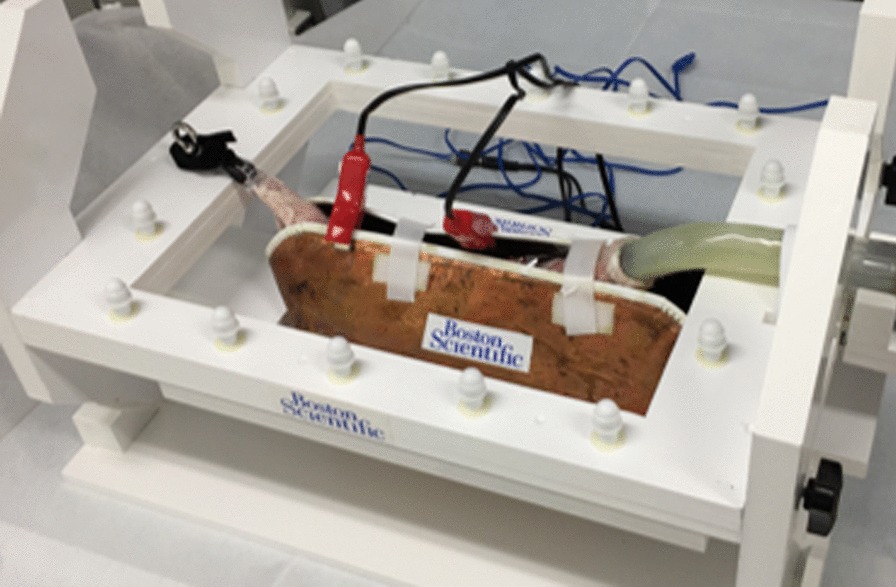
The ex vivo porcine model used for ESD training

**Fig. 3:**
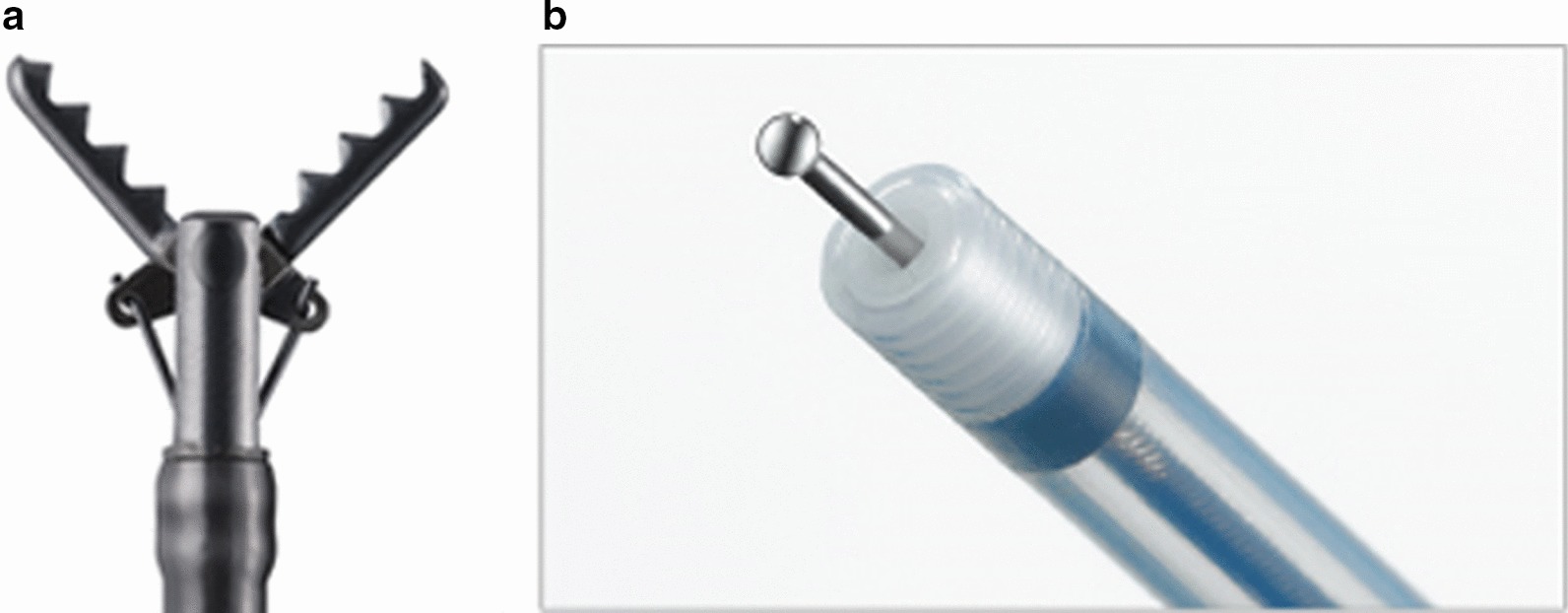
The images of the endo-knives used in the study. **a** The Clutch Cutter: a scissor-type knife. **b** The Flash knife: a needle-type knife

Harvested pig stomachs and esophagi were used as the ex vivo porcine models for ESD. These were frozen for transport and thawed prior to the seminar (shown in Fig. 2). The gastric lumen was cleaned by water lavage, and the stomach was fixed on a plastic tray. An overtube (TOP Co., Tokyo, Japan) was attached to the esophagus, and the pylorus of the stomach was tied. Upper GI endoscopes (EG-450-RD5; Fujifilm, Tokyo, Japan, GIF-Q260J, Olympus, Tokyo, Japan) fitted with disposable straight sift distal attachments [D-201-11804 (Olympus) or Elastic touch (TOP Co.)] were used in the ESD procedure. VIO3 (ERBE, Tubingen, Germany) was used as an electrosurgical power source. The marking was performed in the Forced Coag 9.0 mode, while mucosal incision and submucosal dissection were performed in the endo-cut I, endo-cut 2, or Forced Coag 9.0 modes. Two types of endoscopy rooms were prepared: one for ESD-S and the other for ESD-N. The Clutch Cutter^®^ (DP2618DT, Fujifilm) and Flash Knife (Fujifilm) were used as the scissor-type knife and the needle-type knife, respectively (shown in Fig. 3a, b). A mixture of hyaluronic acid and normal saline with a small amount of indigo carmine was used as the injection solution.

### Endoscopic submucosal dissection procedure

The ESD procedure consists of the following steps: (1) marking around the lesion, (2) lifting the lesion by submucosal injection, (3) making a circumferential mucosal incision outside the marking, and (4) dissecting the submucosal layer. The same procedure was utilized in this study. Prior to ESD, the experts created mock lesions measuring 20 mm in diameter using eight marking dots. All subsequent procedures were performed by the trainees. A traction method using dental floss and a hemoclip (DFC) was used in all cases. After circumferential mucosal incision, the DFC was attached to the edge of the mucosal flap, and the floss was pulled. After confirming good visualization of the submucosal layer, dissection was initiated. The time required to attach the DFC was excluded from the procedure time. The resected specimen was fixed on a plate with proper tension, and the diameters of the mock lesion and the resected specimen were measured using a ruler.

### Definition

The total procedure time was defined as the time from creating the mucosal incision to completion of submucosal dissection. The lesion location was classified into the upper, middle, and lower thirds of the stomach. Furthermore, the lesion position was categorized on the basis of its location in the anterior wall, posterior wall, lesser curvature, and greater curvature [17]. En bloc resection was defined as the resection of the specimen intact with all marking dots. Self-completion was defined as the completion of en bloc resection by the trainee without expert intervention. The operator would be changed if the ESD could not be completed within the preset procedure time of 30 min, the supervisors considered the procedure difficult for the trainees to complete by themselves, or intraoperative perforation occurred. Perforation was defined as a wall defect directly visible on the outside of the stomach. Muscular injury was defined as the exposure and damage of the muscle layer. The difficulty of ESD for trainees was defined as non-self-completion, non-en-bloc resection, perforation, muscular injury, or long procedure time exceeding 20 min.

### Outcome

The primary outcome was the procedure time for ESD. The secondary outcomes included the mucosal incision time, submucosal dissection time, self-completion rate, en bloc resection rate, and rates of complications (muscular injury and intraoperative perforation). These outcomes were compared between the ESD-S and ESD-N groups.

In subgroup analysis, the predictive factors associated with the difficulty of ESD for trainees were investigated. Tumor location (upper/middle third in the stomach or lower), tumor position (greater curvature of the stomach wall or others), endo-knife (ESD-S or ESD-N), and experience of trainees (J-Trainee or S-Trainee) were included among the investigated factors.

### Statistical analysis

All statistical analyses were performed using EZR ver. 1.27 (Saitama Medical Center, Jichi Medical University, Japan) [18]. Continuous valuables were expressed as medians with interquartile ranges (IQR) and analyzed by the student’s t test after logarithmic transformation. Categorical valuables were expressed as numbers with percentages (%) and analyzed by the Chi-square test or the Fisher’s U test. Logistic regression analysis was used to calculate univariate and multivariate-adjusted odds ratios (ORs) and the 95% confidence intervals (95% CIs). In multivariate analysis, OR was adjusted for potentially confounding factors including tumor location, tumor position, endo-knife type, and experience of trainees. Statistical significance was considered as p < 0.05.

## Results

The characteristics and treatment outcomes in the J-Trainees are shown in Table 1. There were no significant differences in the baseline characteristics (such as the location and the size of the specimen) between the ESD-S and ESD-N groups. The total procedure time and mucosal incision time were significantly shorter in the ESD-S group than in the ESD-N group [total procedure time: 16.5 min (range 10.0–31.0 min) vs. 22.3 min (range 10.0–38.0 min), p = 0.018; circumferential incision time: 10.0 min range 6.0–16.0 min) vs. 17.0 min (range 5.0–31.0 min), p = 0.019]. The self-completion rate did not differ significantly between the two groups [(92.9% (13/14) vs. 64.3% (9/14), p = 0.16]. The reason for changing the operator in all cases of expert intervention was the procedure time exceeding 30 min.

**Table 1:** Characteristics and treatment outcomes of ESD between the two groups in J-trainee

	ESD-S (n = 14)	ESD-N (n = 14)	P value
Tumor location (n)			
Upper/middle/lower	1/7/6	1/8/5	0.16
Anterior wall/posterior wall/lesser curvature/greater curvature	5/1/1/7	4/2/2/6	0.18
Specimen size, mm (range)	24.0 (18–41)	26.0 (12–42)	0.96
Median procedure time, min (range)			
Total	16.5 (10–31)	22.3 (10–38)	0.02
Mucosal incision	10.0 (6–16)	17.0 (5–31)	0.02
Submucosal dissection	5.0 (1.5–10)	5.5 (3–17)	0.68
Self-completion, %	13 (92.9%)	9 (64.3%)	0.16
En-bloc resection, %	14 (100%)	14 (100%)	0.25
Complication, %	0 (0%)	2 (14.3%)	0.25
Perforation, %	0 (0%)	0 (0%)	1.0
Muscularis layer injury, %	0 (0%)	2 (14.3%)	0.25

P value was calculated using χ^2^ test or Fisher’s exact test for categorical data

P value was calculated using Student’s t test for continuous data

*ESD* endoscopic submucosal dissection, *J-Trainee* junior-trainee with the experience of less than 1000 endoscopies, *ESD-S* endoscopic submucosal dissection with scissor-type knife, *ESD-N* endoscopic submucosal dissection with needle-type knife

The characteristics and treatment outcomes in the S-Trainees are shown in Table 2. Between the ESD-S and ESD-N groups, there were no significant differences in the total procedure time [18.0 min (9.0–27.0 min) vs. 17.8 min (7.0–31.0 min), P = 0.75], mucosal incision time [12.0 min (5.5–20.0 min) vs. 13.0 min (4.5–17.0 min), P = 0.79], and submucosal dissection time [6.5 min (1.5–10.0 min) vs. 4.8 min (2.5–14.0), P = 0.29]. The self-completion rate did not differ significantly between the two groups [100% (8/8) vs. 87.5% (7/8), p = 1.0]. The reason for changing the operator in all cases of expert intervention was the procedure time exceeding 30 min.

**Table 2:** Characteristics and treatment outcomes of ESD between the two groups in S-Trainee

	ESD-S (n = 8)	ESD-N (n = 8	P value
Tumor location (n)			
Upper/middle/lower	0/4/4	0/3/5	1.0
Anterior wall/posterior wall/lesser curvature/greater curvature	3/1/1/3	2/2/2/2	0.26
Specimen size, mm (range)	37.5 (28–53)	33.5 (25–53)	0.52
Median procedure time, min (range)			
Total	18.0 (9–27)	17.8 (7–31)	0.75
Mucosal incision	12.0 (5.5–20)	13.0 (4.5–17)	0.79
Submucosal dissection	6.5 (1.5–10)	4.8 (2.5–14)	0.29
Self-completion, n (%)	8 (100%)	7 (87.5%)	0.12
En-bloc resection, n (%)	8 (100%)	8 (100%)	1.0
Complication, n (%)	0 (0%)	0 (0%)	1.0
Perforation, n (%)	0 (0%)	0 (0%)	1.0
Muscularis layer injury, n (%)	0 (0%)	0 (0%)	1.0

P value was calculated using χ^2^ test or Fisher’s exact test for categorical data

P value was calculated using Mann–Whitney U test for continuous data

*ESD* endoscopic submucosal dissection, *S-Trainee* senior-trainee with the experience of over 1000 endoscopies, *ESD-S* endoscopic submucosal dissection with scissor-type knife, *ESD-N* endoscopic submucosal dissection with needle-type knife

Regarding complications, no perforation was observed in any case. While muscular injury occurred in two cases treated by J-Trainees in the ESD-N group, no injury occurred in the ESD-S group.

In subgroup analysis, we evaluated the factors associated with the difficulty of ESD (Table 3). The multivariate logistic regression analysis showed that ESD-N (OR 5.28, 95% CI 1.25–22.30; *p* = 0.024) was an independent predictive factor associated with the difficulty of ESD; other factors such as location, position, and experience of trainees were not associated with the difficulty of ESD.

**Table 3:** Predictive factors associated with difficulty of ESD performed by trainees

	Univariate			Multivariate^a^		
	OR	95% CI	P value	OR	95% CI	P value
Location						
Lower	1	Ref	0.60	1	Ref	0.50
Middle/upper	1.40	0.40–4.0		1.63	0.40–6.64	
Location						
AW/PW/LC	1	Ref	0.58	1	Ref	0.47
GC	1.43	0.41–5.1		1.69	0.41–6.96	
Experience of trainee						
J-Trainee	1	Ref	0.34	1	Ref	0.44
S-Trainee	1.9	0.50–7.58		1.8	0.41–8.02	
Device						
Scissor-type knife	1	Ref	0.03	1	Ref	0.024
Needle-type knife	4.5	1.15–17.7		5.28	1.25–22.3	

P value was calculated by logistic regression analysis

*ESD* endoscopic submucosal dissection, *OR* odds ratio, *CI* confidence interval, *AW* anterior wall, *PW* posterior wall, *LC* lessor curvature, *GC* greater curvature, *S-Trainee* senior-trainee with the experience of over 1000 endoscopies, *J-Trainee* junior-trainee with the experience of less than 1000 endoscopies

^a^Adjusted for all factors in this table

## Discussion

**Fig. 4:**
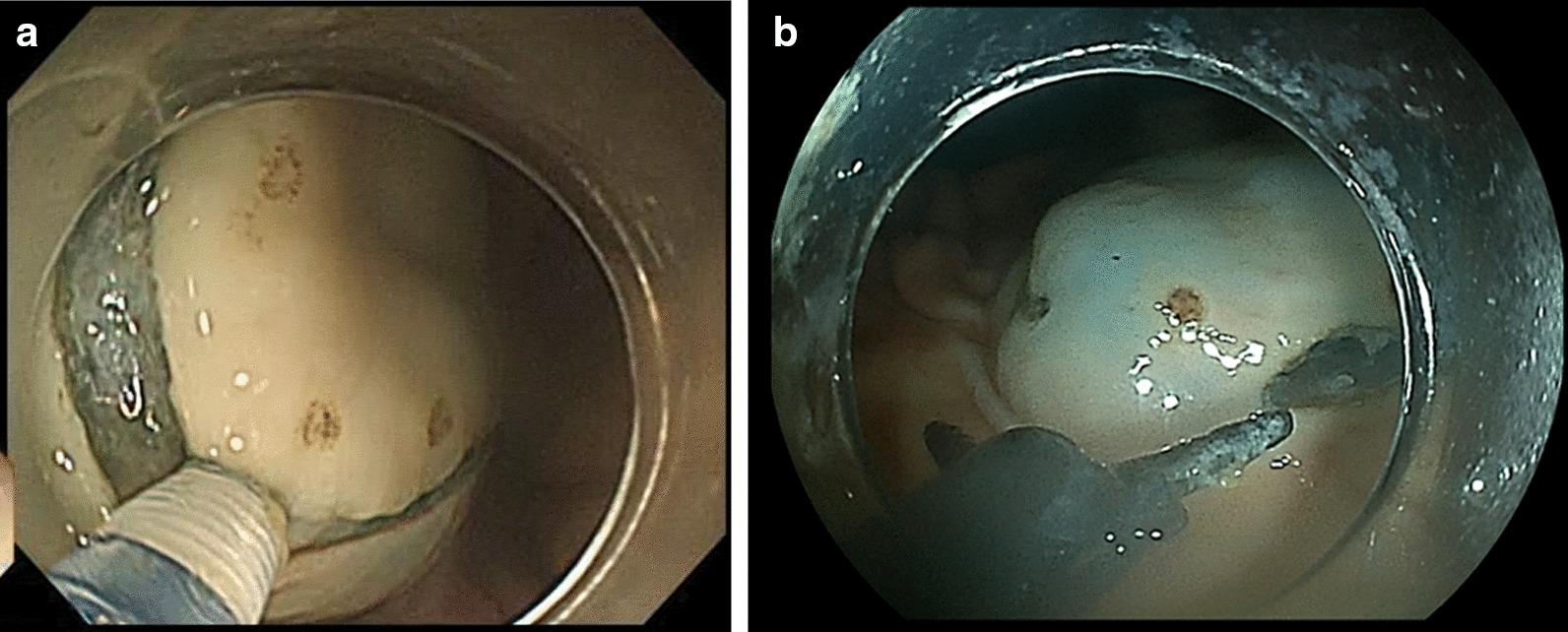
Endoscopic submucosal dissection using a needle-type knife and a scissor-type knife. **a** Mucosal incision using a needle-type knife. **b** Mucosal incision using a scissor-type knife

The J-trainees achieved a significantly shorter procedure time in ESD-S than in ESD-N, although the self-completion rates did not differ significantly between the two groups. The mucosal incision time was also significantly shorter in ESD-S; this may be attributed to the differences in the features of the scissor-type and needle-type knives (Fig. 4). The needle-type knife requires certain operator-specific techniques. The operator moves the endoscope closer to the mucosa, and while maintaining an appropriate distance, applies moderate tension in the direction of the cut [19, 20] (Fig. 4a). Conversely, the scissor-type knife does not require similar techniques. The assistant rotates the endo-knife to adjust the cutting line, and then grasps the target tissue and cuts it [21–24] (Fig. 4b). The biopsy technique used during screening endoscopy allows mucosal incision. ESD-S does not require an advanced technique for manipulating the endoscope; this may contribute to the reduction in the procedure time, especially in the mucosal incision time. Regarding the submucosal dissection time, no significant differences were observed between ESD-S and ESD-N, indicating that differences in the endo-knife type may not affect the outcomes of submucosal dissection. Furthermore, the DFC traction method was applied to all cases to simplify the submucosal dissection procedure; this may have reduced the difference in the treatment outcomes between the two groups [25–27].

Regarding complications, muscular injury occurred in two cases of ESD-N; however, no complications occurred in ESD-S. In ESD-S, the scissor-type knife allows the operator to confirm the cutting line after grasping the tissue; an accidental grasp of the muscle layer can be released prior to cutting, thereby preventing muscle injuries. A previous study also reported lower complication rates in ESD-S than in ESD-N [28]. ESD-S offers an advantage in that experts can confirm the cutting line while teaching the ESD procedure to trainees.

This study was a non-randomized trial. There might be confounding bias that affects the treatment outcomes. Therefore, predictive factors associated with the difficulty of ESD treated by trainees were also evaluated using multivariate analysis, which adjusted and reduced such bias. As a result, ESD-N was an independent predictive factor for the difficulty of ESD. Other factors, including tumor location, position, and experience of trainees, were not independent factors. Although there was no significant difference in procedure time between both endo-knives in S-trainees, considering the result of this multivariate analysis and the fact that self-completion was achieved in all cases of ESD-S, the selection of the scissor-type knife is desirable in S-trainees as well as in J-trainees.

Few reports have compared the treatment outcomes of ESD-S performed by trainees with those of ESDs performed by trainees using other endo-knives [29–31]. Nevertheless, the superiority of the scissor-type knife remains controversial. Dohi et al. conducted a retrospective study to compare the treatment outcomes of gastric ESD between the Clutch Cutter (the scissor-type knife) and Insulate-tip 2 (IT2) knife (KD-611L; Olympus). The use of the Clutch Cutter significantly improved the self-completion rate of ESD in the non-expert group (61.7% in Clutch Cutter vs. 24.5% in IT2, P < 0.001) [29]. Yamashita et al. reported that in colorectal ESDs performed by two trainees, the SB Jr (Sumitomo Bakelite) achieved a greater improvement in the self-completion rate than the Flush Knife (63% vs 39%, P = 0.03) [31]. Conversely, Nagai et al. reported no significant differences in the self-completion rates between the Clutch Cutter and the IT2 knife (66% vs 77%, P = 0.187) for gastric ESD performed by three trainees [30]. Although, with respect to actual clinical practice, these studies include a small number of trainees, with most ESDs being performed at a single center. A small number of trainees renders the study results strongly dependent on the baseline skill and learning curve of the trainees. Furthermore, most endoscopists had already gained experience in gastric and esophageal ESD during the study of colorectal ESD. Therefore, the study outcomes may depend on their experience with specific endo-knife types in such gastric and esophageal ESDs. If the ESD experience among trainees increases, the procedural skill stabilizes, and the endoscopist can no longer be considered a trainee. A previous study reported that trainees could perfect their skills by performing 30 cases of ESD in the lower third of the stomach [32]. Therefore, an evaluation of the treatment outcomes of ESD performed by many trainees at the same baseline may clarify the usefulness of each endo-knife for these trainees.

This study has the following strength: It included 22 trainees from 13 institutions, which is greater than the number of participants included in previous studies. Furthermore, all participants were trainees who had experienced no or less than five ESDs. They were divided into two groups based on their experiences, which enabled a comparison among endoscopists with the same endoscopic skills.

However, this study also has several limitations. First, this was a non-randomized, retrospective study, with a different and limited number of trainees included in the two groups. Multivariate logistic regression analysis was added to reduce such bias. Second, the endoscopists included were mostly Japanese, although two Chinese endoscopists also participated in the study. ESD is widespread in both Japan and China; therefore, the findings of this study may not apply to Western endoscopists that are unfamiliar with ESD. Third, the total procedure time in non-self-completion cases also included the duration in which the experts performed the dissection; our protocol permits experts to take over if the total procedure time exceeds 30 min. Six of the seven non-self-completion instances occurred in ESD-N. In all non-self-completion cases, the experts assisted the trainees in the submucosal dissection. Therefore, a purely-trainee procedure time could not be evaluated. Fourth, this was an ex vivo animal model study. No intraoperative bleeding occurred during the procedure; however, this scenario may differ in ESDs performed in humans. Hemostasis for bleeding is also a technically difficult procedure for trainees and may affect the treatment outcomes. Finally, all ESD cases were assisted by an expert, and the technical skill of the expert may affect the treatment outcomes of ESD-S; if a trainee had assisted with the ESD, the results would have been different. A multi-center prospective study with many trainee endoscopists in an actual clinical setting is warranted to compare the efficacy and safety of ESD between different-type of endo-knives in the future.

## Conclusion

To the best of our knowledge, this study is the first to compare the treatment outcomes of ESD performed by trainees in a porcine model according to the type of endo-knife used. This study revealed that when performing ESD, trainees who have had less experience with endoscopies should select the scissor-type knife.

## Abbreviations

ESD: Endoscopic submucosal dissection
ESD-S: Endoscopic submucosal dissection with a scissor-type knife
ESD-N: Endoscopic submucosal dissection with a needle-type knife
S-Trainee: Senior trainee
J-Trainee: Junior trainee
GI: Gastrointestinal
DFC: Dental floss and a hemoclip
IQR: Interquartile ranges
SD: Standard deviation
IT2: Insulate-tip

## Availability of data and material

The datasets used and analyzed during the current study are available from the corresponding author on reasonable request.
